# Development and Validation of Multiplex Assays for Lupus Nephritis Activity Biomarkers

**DOI:** 10.1016/j.ekir.2025.04.013

**Published:** 2025-04-21

**Authors:** Ellen M. Cody, Alyssa Sproles, James Rose, Bin Huang, Prasad Devarajan, Hermine I. Brunner, Sherry Thornton

**Affiliations:** 1 Division of Pediatric Nephrology, Department of Pediatrics, Medical College of Wisconsin, Milwaukee, Wisconsin, USA; 2 Division of Rheumatology, Cincinnati Children’s Hospital Medical Center, Cincinnati, Ohio, USA; 3 Division of Nephrology and Hypertension, Cincinnati Children's Hospital Medical Center, Cincinnati, Ohio, USA; 4 Division of Biostatistics and Epidemiology, Cincinnati Children's Hospital Medical Center, Cincinnati, Ohio, USA; 5 Department of Pediatrics, University of Cincinnati, Cincinnati, Ohio, USA

**Keywords:** ELISA, Luminex, lupus, MSD, multiplexing, nephrology

## Abstract

**Introduction:**

We aimed to develop multiplex (MLP) assays of 6 biomarkers, namely adiponectin, neutrophil gelatinase-associated lipocalin (NGAL), monocyte chemoattractant protein-1 (MCP-1), kidney injury molecule-1 (KIM-1), ceruloplasmin, and hemopexin used in the Renal Activity Index for Lupus (RAIL) and establish MLP assays using the Milliplex MLP and the electrochemiluminescence Mesoscale Discovery (MSD) technology, to compare with the gold standard of established single immunoassays.

**Methods:**

A total of 104 banked urine samples from the CCHMC Lupus Cohort were used. RAIL biomarker concentrations were assayed using established individual immunoassays, and concentrations were compared with MLP reagents using both the MSD and MLP platforms. MLP assay development involved assessment of biomarker concentrations in 40 individual urine samples, followed by evaluation of optimal sample dilution using 14 additional samples on each platform. Then 50 samples were assayed in duplicate under optimized MLP conditions, and biomarker concentration compared with those using single assays. After correcting for urine creatinine, RAIL scores of the samples were determined and compared between testing platforms (single immunoassays, MLP).

**Results:**

Our results indicate that a 1:25 urine dilution was optimal when using the MLP platforms. Biomarker concentrations by single immunoassays correlated with those on the Milliplex platform strongly for KIM-1, MCP-1, and NGAL (*r* = 0.726–0.86, *P* < 0.0001), moderately for adiponectin (*r* = 0.629, *P* < 0.0001) and weakly for ceruloplasmin (*r* = 0.367, *P* = 0.009). Using the MSD platform, comparatively lower correlations with those by single immunoassay were observed (NGAL: *r* = 0.516, *P* = 0.0001; adiponectin and hemopexin: *r* ≤ 0.29, *P* = 0.042; ceruloplasmin, KIM-1, and MCP-1: all *r* < 0.2).

**Conclusion:**

Milliplex technology is suitable to measure RAIL biomarker concentrations in urine samples diluted 1:25.

## Graphical abstract

**Figure:**
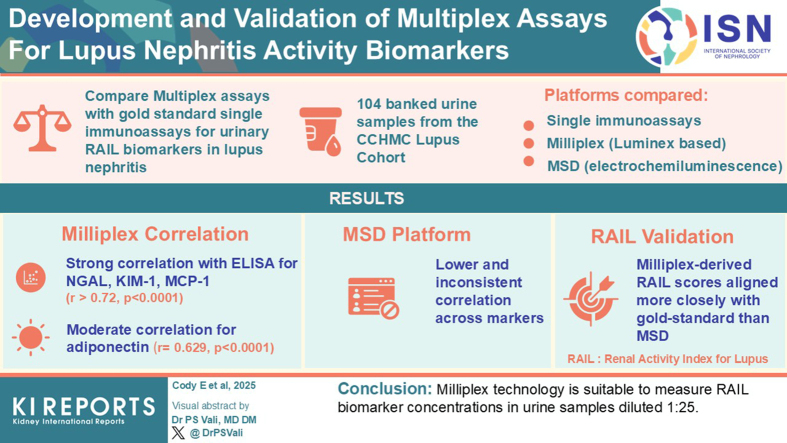

Urine biomarkers are an area of interest for lupus nephritis (LN), because of the inadequacy of current blood tests in diagnosing and monitoring kidney involvement in systemic lupus erythematosus.^1^^,^^2^ The RAIL was first described as a potential urinary biomarker score to capture LN activity in children with LN.^3^ The RAIL score considers the urine concentrations of NGAL, MCP-1, KIM-1, ceruloplasmin, adiponectin, and hemopexin. A higher RAIL score reflects a higher degree of renal inflammation as defined by the National Institutions of Health-Activity Index with changes of RAIL scores shown to correlate with and even anticipate the future course of LN.^3^, ^4^, ^5^, ^6^ A RAIL algorithm similar to that used for pediatric samples has been described for use in adults with LN, yielding comparable accuracy for detecting LN activity and course.^7^

Currently, individual RAIL biomarkers are assayed using individual single immunoassays, primarily enzyme-linked immunosorbent assay (ELISA). Besides being more time and labor intensive, such single assays necessitate the use of larger amounts of sample, limiting the use of the RAIL in clinical practice and in support of research.^8^ MLP assays allow for measuring several analytes with 1 assay run, offering the potential to reduce assay cost, as well as create a more rapid turnaround time for estimating concentrations of several analytes that are needed to inform medical decision making or evaluation of disease status and response to treatment. This will reduce labor, require fewer testing plates, and minimize run times, as well as decrease sample volume required for testing. Several examples for MLP assays particularly in regard to cellular responses have been established, including most notably those for detecting SARS-CoV-2 antigens and antibodies.^9^, ^10^, ^11^, ^12^, ^13^, ^14^

ELISAs for cytokines and other analytes are available using MLP testing platforms offered by several vendors. Among the more established and more widely used testing platforms that offer the potential of assaying several analytes with 1 assay run, are those offered by Millipore (MilliporeSigma, Burlington, MA) and Meso Scale Discovery (MSD, Rockville, MD).

The objectives of this study were as follows: to (i) develop novel bead-based MLP assays of the 6 biomarkers that are included in the RAIL, that is, adiponectin, NGAL, MCP-1, KIM-1, ceruloplasmin, and hemopexin; (ii) compare concentrations of the RAIL biomarkers when measured using the MLP and single ELISAs; and (iii) evaluate the impact of the biomarker measurement approach on the RAIL score. Herein, we used bead-based assays for use on Millipore’s Milliplex platform and the electrochemiluminescence immunoassay platform from MSD.

## Methods

**Figure 1:**
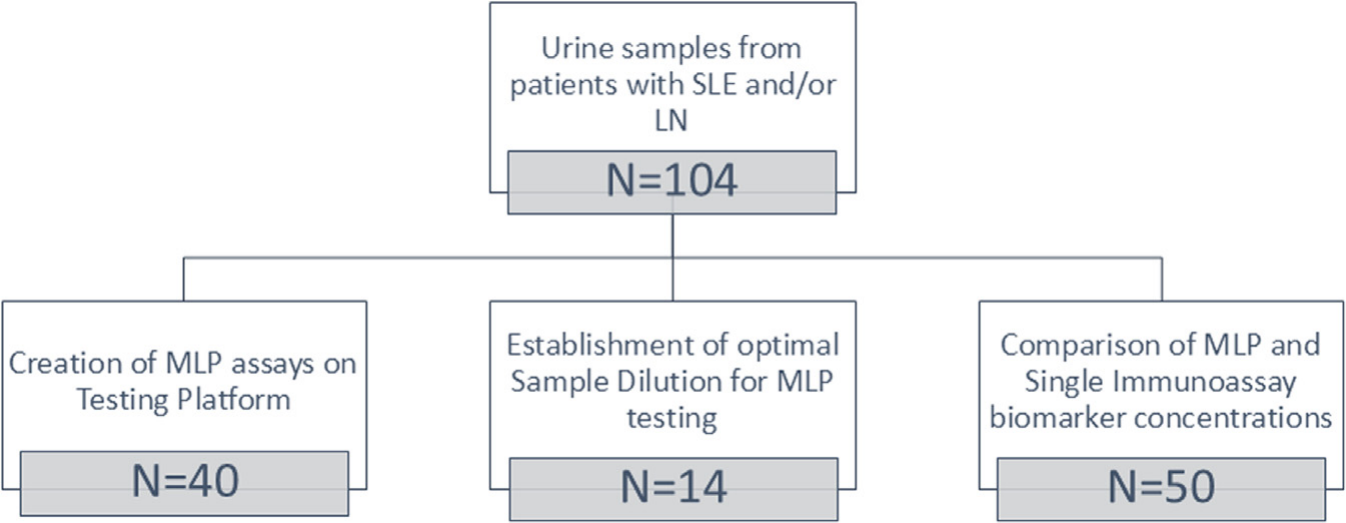
Flow diagram of urine samples used in the study.

This was a retrospective analysis performed on banked urine samples that were obtained from the CCHMC Lupus Database and the biorepository (IRB 2008 0634). A total of 104 unique urine samples were used from patients with systemic lupus erythematosus^15^ with or without kidney involvement,^16^ that is, LN (Figure 1). After collection, urine samples were spun for 10 minutes at 1600 RPM before storage without the pellet at −80 °C until used in this study. All 104 samples were assayed using single immunoassays. To develop the MLP assays (Millipore, MCS), 40 samples were used to assess levels of the 6 analytes initially using dilutions of 1:5 and 1:100. Then, another 14 samples were used to confirm optimal sample dilution for MLP testing. Finally, 50 samples were used to assess the reliability of and compare biomarker concentrations across the 3testing platforms (single ELISAs, MSD, Milliplex). These 50 urine samples were selected to cover the range of RAIL biomarker concentrations in our prior research.^3^^,^^6^, ^7^, ^8^

### Single Assays for the RAIL Biomarkers

Human MCP-1 was measured using commercially available ELISA (R&D Systems, Minneapolis, MN, DCP00), dilution 1:1, with a mean minimal detectable dose of 1.7 pg/ml. Intraassay and interassay were 5% and 5.1%, respectively. Human adiponectin was measured using commercially available ELISA kit (R&D system, Minneapolis, MN, DRP300), run undiluted, with a mean minimal detectable dose of 0.246 ng/ml. Intraassay and interassay coefficients of variation were 3.7% and 6.8%, respectively. Human ceruloplasmin was measured using ELISA Kit (Assaypro LLC, St. Charles, MO, EC4201-1), diluted 1:50, with a mean minimal detectable dose of 0.087 ng/ml. Intraassay and interassay coefficients of variation were 4.9% and 9.8%, respectively. Human hemopexin was measured using commercially available ELISA kit (Assaypro LLC, St. Charles, MO, EH2001-1), dilution 1:20, with a mean minimal detectable dose of 4.2 ng/ml. Intraassay and interassay coefficients of variation were 4.7% and 9.2%, respectively. Human KIM-1 was measured using commercially available ELISA kit (R&D System, Minneapolis, MN, DKM100), run undiluted, with a mean minimal detectable dose of 0.009 ng/ml. Intraassay and interassay coefficients of variation were 4.2% and 6.7%, respectively. A Roche Cobas c 311 clinical chemistry analyzer, using a commercially available assay (BioPorto, Denmark, Catalog KIT ST001RA for NGAL; Roche Diagnostics, Indianapolis, IN, Reference 03263991190 for creatinine), was used to measure both human NGAL and urine creatinine. NGAL had a lower limit of detection of 9.8 ng/ml and creatinine had a lower limit of detection of 1.1 mg/dl. Concentrations of each of the RAIL biomarkers are reported in ng/ml except for MCP-1 in pg/ml and creatinine in mg/dl. These gold standard assays were primarily tested using traditional ELISA whereas NGAL was tested with this assay thus, these single assays are referred to as “single immunoassays.”

### Step 1: Set-Up of 6-Plex for RAIL Assays

Milliplex bead assays use fluorescently coated beads based on Luminex technology. Analyte-specific capture antibodies are bound to these beads, and then biotinylated detection antibodies are added. Streptavidin-phycoerythrin is added, which binds to the biotinylated detection antibodies, and is read by a flow cytometry-based system, quantified based on fluorescent reporter signals. Milliplex bead assays were available for 5 of these markers, namely adiponectin, MCP-1, KIM-1, ceruloplasmin, and NGAL. Development of a hemopexin single Milliplex bead assay was done by MilliporeSigma to determine the best antibody available for binding and lowest background within urine. After this was done, it was multiplexed with the other 5 analytes for the assay and additional testing was performed to determine the sensitivity and specificity of the MLP assay relative to recombinant standards. After receiving the 6-plex assay from Millipore, we assessed concentrations of the RAIL biomarkers in 40 unique urine samples at both a 1:5 and 1:100 dilution to determine whether the values for the analytes were included within the limits of the standard curves. In addition to performing single immunoassays, side by side, the same 40 unique samples were assayed using after 1:5 and 1:100 dilution on a custom 6-plex assay from MSD. The MSD platform uses electrochemiluminescent labels conjugated to antibodies. Electricity is applied to the plate which leads to light emission, and light intensity is measured to quantify analytes in the sample (MSD, Rockville, MD).

### Step 2: Determination of the Optimal Sample Dilution

To test for the optimal dilution of the urine sample for the assay, 14 different samples that had not previously been subjected to freeze-thaw were assessed for concentrations of the 6 biomarkers using the 6-plex. The samples were diluted 1:25, 1:100, 1:500, and 1:10,000 and assessed for protein concentrations across all 3 platforms (gold standard ELISA, the Milliplex, and the MSD) and tested using single immunoassays.

### Step 3: Comparison of 6-plex Assays With Samples Tested Previously by ELISA

We compared RAIL biomarker concentrations determined by the 6-plex assays with those determined by single Immunoassays (gold standard) using another 50 unique urine samples that had been subjected to another previous freeze-thaw cycle for the performance of single assays for the RAIL biomarkers. These samples were diluted 1:25 and assessed in duplicates using the Milliplex and MSD platforms. The samples were selected to cover the range of RAIL biomarker concentrations in patients with LN as observed in our previous studies.^3^^,^^6^, ^7^, ^8^

### Statistical Analysis

Prior to use in the RAIL algorithm, biomarker concentrations were natural log–transformed. The adult RAIL algorithm was used as previously established, with standardized and nonstandardized scores.^7^ The standardized RAIL score refers to the score that used creatinine standardized urinary biomarkers, whereas the nonstandardized score used absolute values of the urinary biomarkers.

For Step 1, Bland-Altman plots were constructed to assess the relationship of biomarker concentrations by MLP measurement with those by single immunoassays across a wide range of urine biomarker quantities. Guided by our initial experiments in Step 1, we then moved to Step 2, which was to establish the most suitable urine dilution to best estimate all individual RAIL biomarkers reliably. We compared the concentration of each RAIL biomarker, as well as all the calculated standardized and nonstandardized RAIL score, after adjustment for differences in the read-out of biomarker quantities between the single immunoassays and 6-plex assays (Milliplex, MSD). For Step 3, Deming Regression was used to model the relationship between each pair of experimental conditions with the control condition. Pearson correlation coefficients were calculated for each experimental condition against the control, with 0.1 to 0.39 representing weak correlation, 0.4 to 0.69 representing moderate correlation, 0.7 to 0.89 representing strong correlation, and > 0.9 representing very strong correlation.^17^ Two-sided *P*-value < 0.05 was considered statistically significant. All statistical analysis was done via SAS Software, Version 9.4, (Cary, Indiana).

## Results

### Set-Up of the 6-Plex Assays (Step 1)

**Figure 2:**
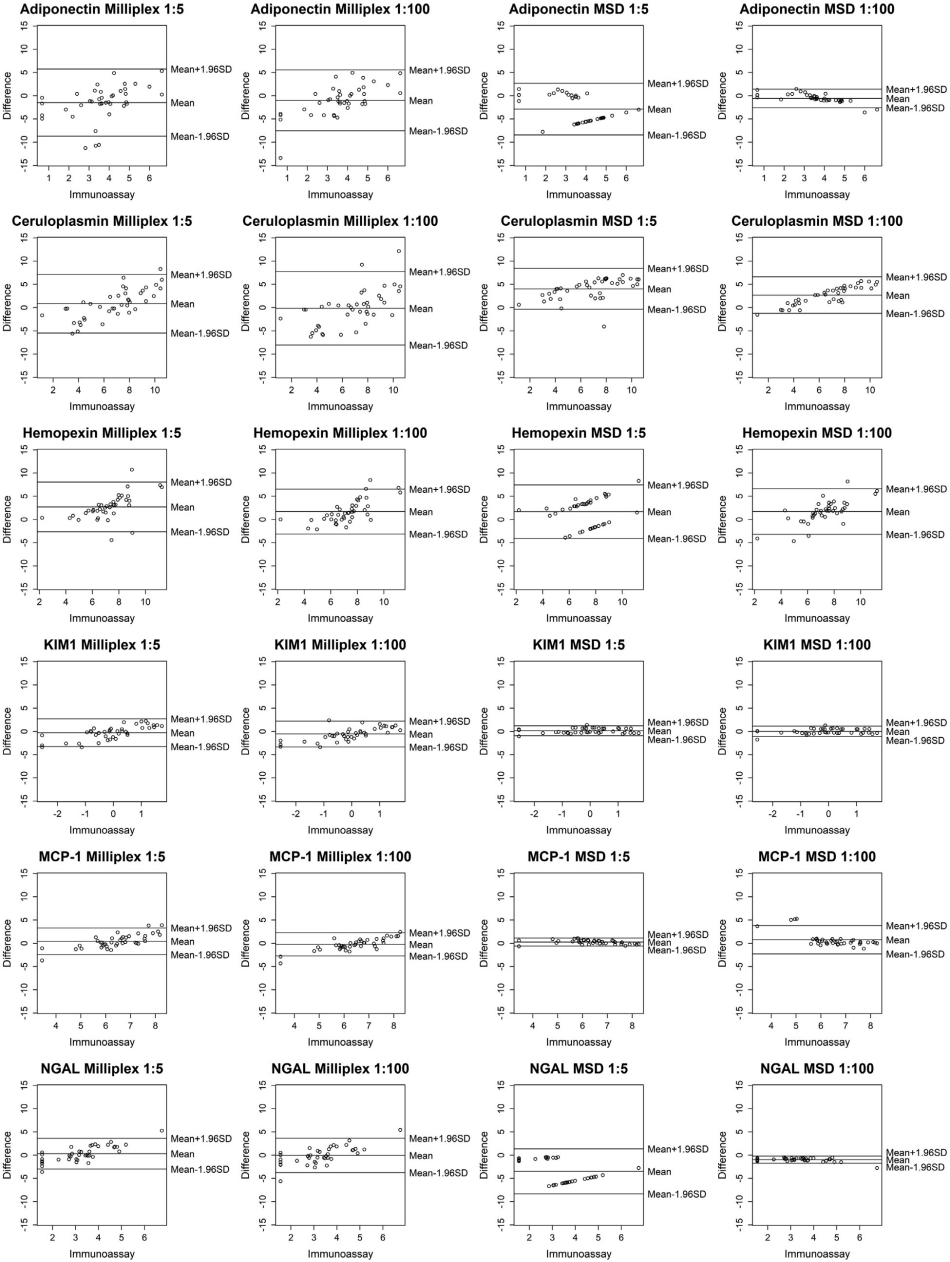
Bland-Altman plots comparing each value of multiplex assay (Milliplex then MSD at 1:5 and 1:100, respectively) against gold standard immunoassay for each biomarker. KIM-1, kidney injury molecule-1; MCP-1, monocyte chemoattractant protein-1; MSD, Mesoscale Discovery; NGAL, neutrophil gelatinase-associated lipocalin.

The 40 individual sample results of the custom 6-plex (both Milliplex and MSD) showed that the 1:100 dilution provided the most consistent data for both assays over the 1:5 dilution but was still not the optimal dilution. The Bland-Altman plots (Figure 2) depict that the measurements of most RAIL biomarkers remain within the 95% confidence interval (± 1.96 SD) of the measurements by the respective single immunoassays. For the Milliplex assay, the 1:100 dilution provided the most reproducible data; however, select samples diluted to 1:100 were not in the range of the standard (Figure 2). Similarly to Milliplex assay, the MSD assay provided reproducible data when compared with single immunoassays for both 1:5 and 1:100 dilutions, although there were also some samples not in range of the standard (Figure 2).

### Optimal Sample Dilution Results (Step 2)

**Figure 3:**
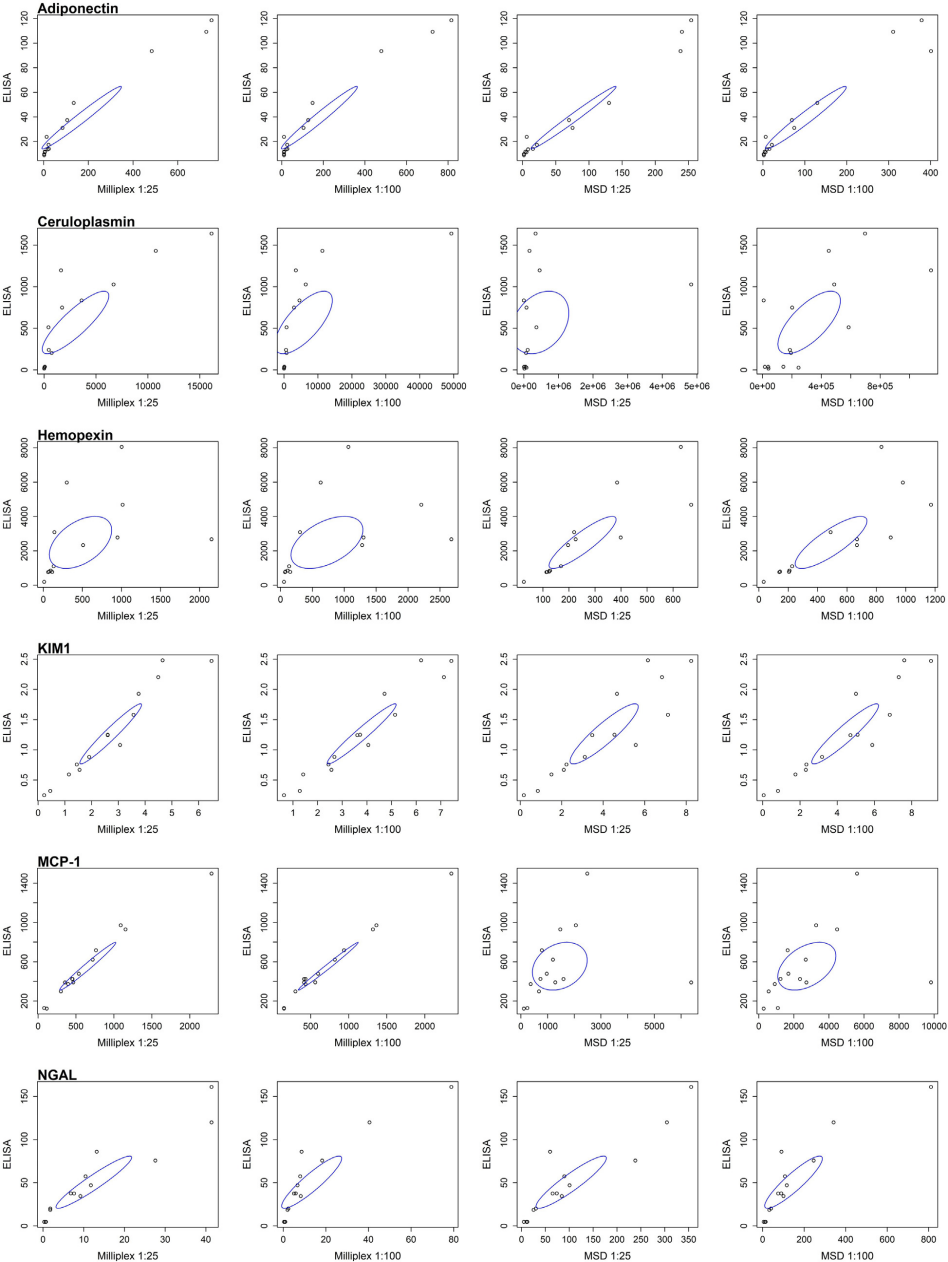
Scatter plots demonstrating correlation coefficients of each multiplex assay (Milliplex and MSD respectively) against the gold standard immunoassay at 1:25 and 1:100, respectively for each biomarker, with the ellipses showing the 95% confidence interval. KIM-1, kidney injury molecule-1; MCP-1, monocyte chemoattractant protein-1; MSD, Mesoscale Discovery; NGAL, neutrophil gelatinase-associated lipocalin.

The 14 samples that were diluted 1:25, 1:100, 1:500, and 1:10,000 revealed that the 1:25 dilution was optimal (Table 1, Figure 3) (data for 1:500 and 1:10,000 not shown). The 1:25 dilution with Milliplex best fit the sample measurement on the measurable part of the standard curves for all 6 analytes, with strong to very strong correlation coefficients of 0.855 to 0.98 for all analytes (*P* < 0.00001), apart from hemopexin with a moderate correlation of 0.465 (*P* = 0.094). The 1:100 dilution with Milliplex overall had slightly lower correlation coefficients for all analytes. The 1:25 MSD dilution demonstrated weak correlation for MCP-1 (0.254, *P* = 0.381) and ceruloplasmin (0.301, *P* = 0.296). The 1:100 MSD dilution demonstrates moderate correlation for ceruloplasmin (0.694, *P* = 0.006) and MCP-1 (0.426, *P* = 0.128).

**Table 1:** Correlation of biomarker concentrations by single immunoassay with those from the 2 multiplex assays using 2 different urine dilutions^a^

Biomarker	Milliplex 1:25 dilution	Milliplex 1:100 dilution	MSD 1:25 dilution	MSD 1:100 dilution
Adiponectin (ng/ml)	0.981, *P* < 0.0001	0.980, *P* < 0.0001	0.987, *P* < 0.0001	0.973, *P* < 0.0001
Ceruloplasmin (ng/ml)	0.855, *P* < 0.0001	0.709, *P* = 0.005	0.301, *P* = 0.296	0.694, *P* = 0.006
Hemopexin (ng/ml)	0.465, *P* = 0.094	0.484, *P* = 0.080	0.884, *P* < 0.0001	0.823, *P* = 0.0003
KIM-1 (ng/ml)	0.965, *P* < 0.0001	0.968, *P* < 0.0001	0.903, *P* < 0.0001	0.936, *P* < 0.0001
MCP-1 (pg/ml)	0.984, *P* < 0.0001	0.992, *P* < 0.0001	0.254, *P* = 0.381	0.426, *P* = 0.128
NGAL (ng/ml)	0.946, *P* < 0.0001	0.910, *P* < 0.0001	0.921, *P* < 0.0001	0.903, *P* < 0.0001

ELISA, enzyme-linked immunosorbent assay; KIM-1, kidney injury molecule-1; MSD, Mesoscale Discovery; MCP-1, monocyte chemoattractant protein-1; NGAL, neutrophil gelatinase-associated lipocalin.

a All statistical variables are Pearson Correlation Coefficients, compared with Gold Standard ELISA.

### Comparison of 6-plex Versus Single Immunoassay Biomarker Concentrations and Intraassay Reliability (Step 3)

**Figure 4:**
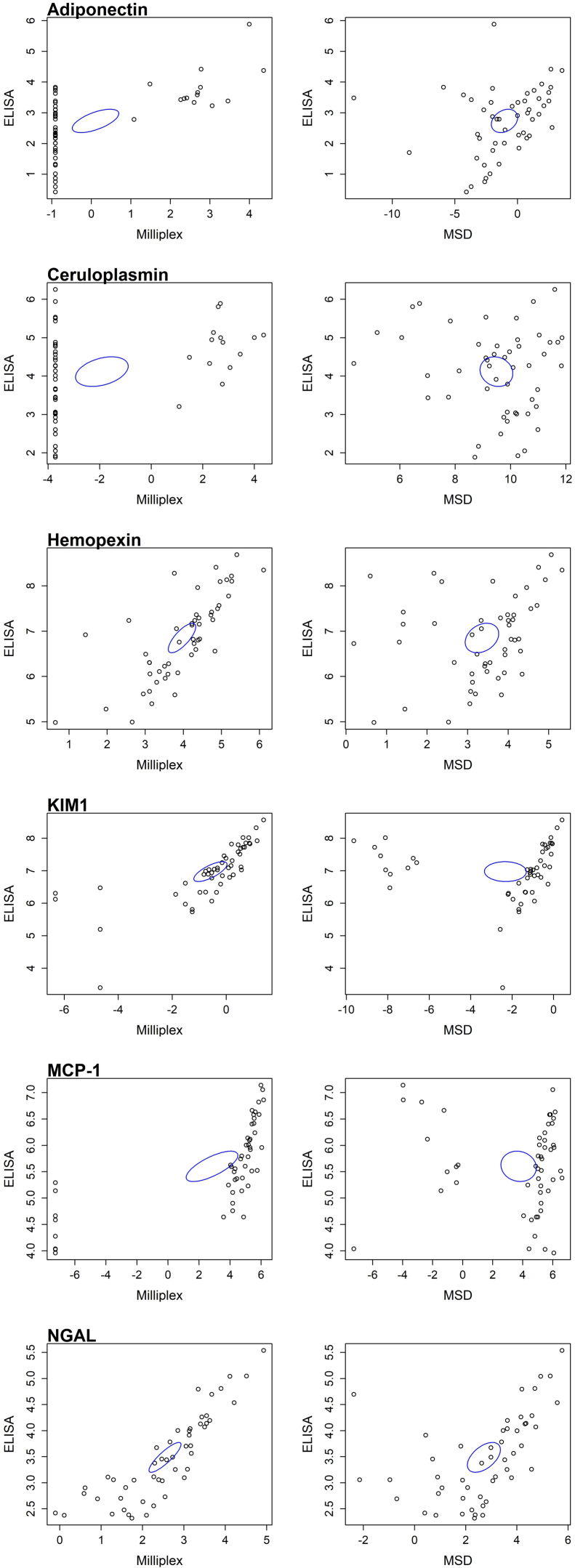
Scatter plots demonstrating the natural log–converted values of Milliplex and MSD respectively in duplicate when compared with gold standard Immunoassay for each biomarker, with ellipses showing 95% confidence interval. KIM-1, kidney injury molecule-1; MSD, Mesoscale Discovery; MCP-1, monocyte chemoattractant protein-1; NGAL, neutrophil gelatinase-associated lipocalin.

Fifty urine samples with known RAIL biomarker concentrations by single ELISAs were assayed via Milliplex and MSD. This was performed after a freeze-thaw cycle, using 1:25 urine dilutions, and results are depicted in Figure 4 and Table 2. Single immunoassay concentrations of RAIL biomarkers were at least moderately associated with those from the Milliplex platform, except for ceruloplasmin. Further biomarker concentrations by Milliplex testing were highly consistent (all *r* ≥ 0.692; all *P* < 0.0001). Conversely, single immunoassay measurements of the biomarker concentrations were at most moderately, but often not significantly, associated with those from the MSD platform MLP assay (*r* = −0.125 to 0.516; *P* = 0.881–0.0001). Nonetheless, the MSD 6-plex assay yielded consistent measurements of biomarker concentrations (all *r* ≥ 0.894; all *P* < 0.0001). NGAL performed the best for both MSD and Milliplex assays, with moderate correlation at 0.516 (*P* = 0.0001), whereas adiponectin and hemopexin had weak correlations between MSD and ELISA (0.289–0.29, *P* = 0.042). Finally, biomarker concentrations from the MSD and Milliplex assays were no more than moderately correlated with each other (Table 2). Specifically, ceruloplasmin showed no correlation (−0.194, *P* = 0.178), adiponectin, KIM-1, MCP-1, and RAIL all showed a weak correlation between the 2 (0.101–0.223, *P* > 0.05). NGAL alone showed a moderate correlation (*r* = 0.649, *P* < 0.0001). Together, the results indicate that the 1:25 Milliplex 6-plex assay performed better than the MSD 6-plex assay.

**Table 2:** Multiplex assays at optimal dilution, correlation to single immunoassay reliability^a^

Biomarker	Milliplex: ELISA	Milliplex: Milliplex	MSD: ELISA	MSD: MSD	Milliplex: MSD
Adiponectin (ng/ml)	0.629, *P* < 0.0001	0.999, *P* < 0.0001	0.290, *P* = 0.042	0.999, *P* < 0.0001	0.178, *P* = 0.217
Ceruloplasmin (ng/ml)	0.367, *P* = 0.009	0.999, *P* < 0.0001	-0.125, *P* = 0.389	0.936, *P* < 0.0001	–0.194, *P* = 0.178
Hemopexin (ng/ml)	0.759, *P* < 0.0001	0.999, *P* < 0.0001	0.289, *P* = 0.042	0.997, *P* < 0.0001	0.418, *P* = 0.003
KIM-1 (ng/ml)	0.732, *P* < 0.0001	0.901, *P* < 0.0001	−0.022, *P* = 0.881	0.999, *P* < 0.0001	0.101, *P* = 0.486
MCP-1 (pg/ml)	0.726, *P* < 0.0001	0.952, *P* < 0.0001	-0.096, *P* = 0.507	0.894, *P* < 0.0001	0.192, *P* = 0.181
NGAL (ng/ml)	0.866, *P* < 0.0001	0.981, *P* < 0.0001	0.516, *P* = 0.0001	0.999, *P* < 0.0001	0.649, *P* < 0.0001

ELISA, enzyme-linked immunosorbent assay; KIM-1, kidney injury molecule-1; MSD, Mesoscale Discovery; MCP-1, monocyte chemoattractant protein-1; NGAL, neutrophil gelatinase-associated lipocalin.

a All statistical variables are Pearson Correlation Coefficients, compared with Gold Standard ELISA.

### Correlation of RAIL Scores Between Biomarker Measurements by Single Immunoassays Compared With MLP Assay

**Figure 5:**
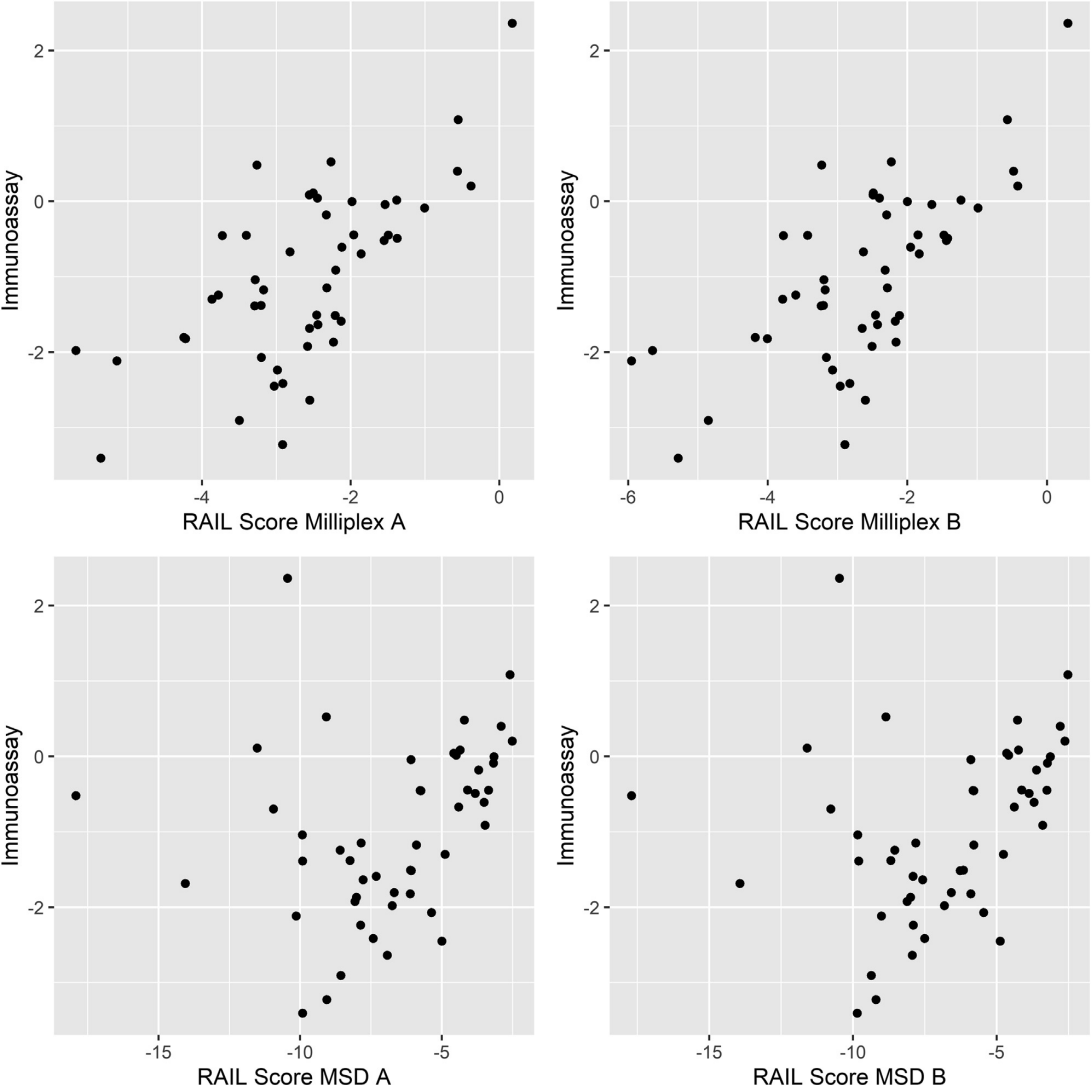
Scatter plots demonstrating the RAIL score calculated using Milliplex and MSD respectively in duplicate when compared with gold standard ELISA. ELISA, enzyme-linked immunosorbent assay; MSD, Mesoscale Discovery.

RAIL scores were then calculated using single immunoassays and Milliplex biomarker concentrations, respectively, with and without standardization for urine creatinine.^7^ As shown in Figure 5*,* RAIL scores from biomarker measurements using the Milliplex methodology had a stronger correlation with the single immunoassays when compared with MSD. Indeed, RAIL scores from Milliplex results also had a stronger correlation to the RAIL score by single immunoassays of (*r* = 0.665; *P* < 0.0001) demonstrating moderate correlation, than when considering biomarker concentrations from the MSD platform (*r* = 0.273; *P* = 0.055), with only a weak correlation.

## Discussion

In this study, we show the feasibility of MLP-based methods for detecting urine biomarkers being evaluated for use in LN. Overall, though absolute values are different by methodology as expected, we report strong correlations for the biomarkers when comparing Milliplex to gold standard ELISA. It should be noted that MSD has increased sensitivity at the lower limits, particularly for adiponectin and ceruloplasmin.

We have previously shown that biomarker concentrations of NGAL, KIM-1, MCP-1, and adiponectin using a 4-plex Milliplex assay correlate well with those using single plex assays.^8^ In this earlier study, hemopexin and ceruloplasmin were difficult to detect, particularly at high concentrations.^18^^,^^19^ With our newly developed study, we now show improved detection in the higher range of measurement for both hemopexin and ceruloplasmin, which is important for determining LN activity using the RAIL score.

Urinary biomarkers continue to be an active area of study in moving diagnosis and management of LN forward, with many under study, including the 6 that are a part of RAIL.^20^ The rheumatology and nephrology communities remain reliant on kidney biopsy to fully assess disease activity and respond to therapy, particularly with multiple new therapies under development.^21^^,^^22^ Individual urinary biomarkers have been used to detect kidney injury during drug development, including in LN; however, a full panel to assess LN activity seems needed to improve the assessment of LN response to therapy, or flare in clinical settings and potentially in support of testing novel LN medications in clinical trials.^23^ With the development of an MLP platform for RAIL, the urine biomarkers may be run using lower volumes of urine sample, with a more rapid turnaround time.^24^^,^^25^ In this study, the total volume of sample used to run the Milliplex was 25 μl, MSD was 25 ul, and the ELISAs were 325 μl. The hands-on time for the ELISAs is 5 hours, compared with 1 hour for Milliplex, and 1.5 hours for MSD.

Given the new methodology on the Milliplex platform and that read-outs of biomarker concentrations are not identical to those from single immunoassays, further studies will be needed to produce an accurate, adapted algorithm to determine the pertinent RAIL values that correlate with inflammation detected on renal biopsy. We have previously shown stability of these 6 biomarkers under various clinical conditions, mimicking storage and deep freeze, as well as potential shipping conditions,^18^^,^^19^ with hemopexin demonstrating the most variability under 1 to 2 times freeze-thaw cycles.

It should be noted that there are several limitations to the current study. A study by our group showed increased variability with freeze-thaw cycles, particularly at the lower limits of detection with increasing freeze-thaws.^18^ There is the need to further address ceruloplasmin degradation when RAIL is measured from samples that have undergone freeze-thaw cycles.

A similar issue may be contributing to the moderate correlation seen with hemopexin. As noted above, in a previous study by our group, hemopexin demonstrated some variability after 1 to 2 times thaw and with longer storage.^18^ This degradation may lead to the difficulty detecting lower limits for both biomarkers, as well as the increased variability. Another potential variable is the custom antibody used for hemopexin in the development of the Milliplex assay. The antibodies used for these assays, more than likely detect different epitopes on a specific analyte and is proprietary information not provided by the manufacturer. Thus, some antibodies potentially may be less reliable and less sensitive in detecting the protein, resulting in the lower concentrations observed in this study.

Overall, this study demonstrates a significant step forward in creating a urine biomarker panel to detect active LN by moving the RAIL score toward clinical utility, with the success in the development of the 6-plex assay. The next steps will be further studies in conjunction with biopsy to establish the pertinent RAIL values that correlate with high National Institutions of Health-Activity Index detected on renal biopsy.

## Disclosure

HI reported funding from FALK medical trust, and a patent in RAIL biomarkers. PD reported funding from FALK medical trust, a patent in RAIL biomarkers, being coinventor on patents using NGAL as a biomarker of kidney injury. PD is the Senior Medical Director of BioPorto, Inc. All the other authors declared no competing interests.
